# Risk Factors related to Late Failure of Dental Implant—A Systematic Review of Recent Studies

**DOI:** 10.3390/ijerph17113931

**Published:** 2020-06-02

**Authors:** Thanh An Do, Hoang Son Le, Yen-Wen Shen, Heng-Li Huang, Lih-Jyh Fuh

**Affiliations:** 1 School of Dentistry, China Medical University, 91 Hsueh-Shih Road, Taichung 40402, Taiwan; dothanhan007@gmail.com (T.A.D.); a2312830@ms28.hinet.net (Y.-W.S.); 2 Department of Oral Surgery, Faculty of Odonto-Stomatology, University of Medicine and Pharmacy at Ho Chi Minh City, 217 Hồng Bàng, Phường 11, Quận 5, Ho Chi Minh City 700000, Vietnam; sonrhm07@gmail.com; 3 Department of Bioinformatics and Medical Engineering, Asia University, 500 Lioufeng Rd., Wufeng, Taichung 41354, Taiwan

**Keywords:** late failure of dental implant, risk factor, PRISMA guideline, patient history, clinical parameters, decisions made by the clinician

## Abstract

Resolving late failure of dental implant is difficult and costly; however, only few reviews have addressed the risk factors associated with late failure of dental implant. The aim of this literature review was to summarize the influences of different potential risk factors on the incidence of late dental implant failure. The protocol of this systematic review was prepared and implemented based on the PRISMA (Preferred reporting items for systematic reviews and meta-analyses) guideline. In December 2018, studies published within the previous 10 years on late dental implant failure were selected by fulfilling the eligibility criteria and the risk factors identified in qualified studies were extracted by using a predefined extraction template. Fourteen eligible studies were assessed. The common risk factors for late failure were divided into three groups according to whether they were related to (1) the patient history (radiation therapy, periodontitis, bruxism and early implant failure), (2) clinical parameters (posterior implant location and bone grade 4) or (3) decisions made by the clinician (low initial stability, more than one implant placed during surgery, inflammation at the surgical site during the first year or using an overdenture with conus-type connection). Clinicians should be cautions throughout the treatment process of dental implant—from the initial examination to the treatment planning, surgical operation and prosthesis selection—in order to minimize the risk of late failure of dental implant.

## 1. Introduction

Dental implants have become a common choice among the treatment options for missing teeth rehabilitation since they were first introduced by Branemark in the 1970s [1]. However, this treatment modality has limitations, with previous reports of failure rates of dental implant ranging from 1% to 19% [2,3]. These failures could be classified into early failure and late failure based on the time when the abutment was connected: early failures occurred before the application of functional loading, and late failures occurred after applying occlusal loading or the first removal of the provisional restoration in cases of immediate implant loading [4].

Early failure represents a failure to establish osseointegration of dental implants, while late failure is the failure of either the established osseointegration or function of dental implants [5]. While early failure is solely biologic complications [6], late failure could have either biologic or mechanical complications. Biologic complications could be due to peri-implantitis, it usually involves the resorption of soft and hard tissue [7,8]. Mechanical complications could be due to improper implant loading design, it could lead to the fracture of implant body, screw body or implant supra-structure [9].

The interval from a diagnosis of dental implant failure to its removal is significantly longer in late failure than early failure, and late failure is also associated with greater bone loss [7]. Moreover, late failure occurs after the final prosthesis has been placed, and so it is more like to lead to patient complaints about cost and the greater effort needed to resolve the condition. These aspects mean that resolving late failure is more difficult. Therefore, identifying risk factors related to late dental implant failure could help in predicting the treatment outcomes and also preventing conflicts not only in the patient–doctor relationship, but also between the surgeon and prosthodontists/restorative specialists.

Previous studies have indicated that peri-implantitis and implant overloading were common risk factors for late failure [7,10], but little is known about other factors affecting the maintenance of osseointegration of implant. In contrast to numerous reviews of the risk factors associated with early failure of dental implant, only one review addressed risk factors associated with late dental implant failure in this decade [8]. Prosthesis overloading, peri-implantitis, and improper fit of the prosthesis were reported as the risk factors associated with late failure [8]. However, that review was likely an author’s commentaries since the methodology not being reported and the lack of other information about the included studies. The aim of the present study was to review the literatures within ten years on the potential factors associated with late failure of dental implant during the functional loading stage by systematic review processes following the PRISMA (Preferred reporting items for systematic reviews and meta-analyses) guideline.

## 2. Methodology

### 2.1. Protocol and Key Question

The protocol of this systematic review was prepared and implemented based on the PRISMA guideline [11]. The purpose of this review was to answer the following question: “What factors could affect the late-failure rates of dental implants?”

### 2.2. Eligibility Criteria

The following criteria were applied to identify eligible articles for inclusion in this review: (1) involving human subjects and (2) the article published within 10 years of the search date. Articles were excluded due to any of the following reasons: (1) reporting on an in vitro study or being a case report, letter to the editor or review; (2) no definitive conclusion reported regarding the late dental implant failure; or (3) placement of implants other than of the dentoalveolar type, such as zygomatic or subperiosteal implants.

### 2.3. Literature Resource and Search Strategy

An electronic search was performed on 1 December 2018 of the PubMed database to retrieve articles published in English during the previous 10 years. The following search string was used for retrieving potential articles: “dental implan* [Mesh] AND late [all fields] AND failur* [all fields]”. This search strategy was applied with the aim of identifying as many relevant publications as possible.

### 2.4. Article Review Process

The searched articles were independently assessed by two reviewers. All of the identified articles’ title were screened to determine the number of Abstracts to be examined. In the next round, article Abstracts were screened for whether the study contents potentially matched the purpose of this review. A consensus was achieved between the reviewers on the qualified list of articles for the next round of the review. The full texts of all eligible articles were then read, and articles that did not fulfill the eligibility criteria were eliminated. The references lists of the articles were manually searched to analyze further relevant studies. A consensus was also achieved at the end of this round between the two reviewers on the final list of articles for further data extraction. Cohen’s kappa was used to estimate the interexaminer reliability.

A Microsoft Excel worksheet was used to collect data extracted from the eligible articles. The following information was extracted: name of the first author, year of publication, study design, number of patients, number of implants placed, number of patients with late-failure implants, number of late-failure implants, follow-up duration and risk factors for late-failure implants. The following characteristics were considered when determining the risk factors: patient demographics (age and sex), overall health status (systemic disease, oral disease, smoking status and oral habits), anatomy-related characteristics (implant location, bone condition, number of remaining teeth and opposing dentition), implant-related characteristics (length, diameter, surface type and brand), surgical procedure and prosthesis aspects (loading protocol, retention type and prosthesis design).

## 3. Results

### 3.1. Study Selection

**Figure 1:**
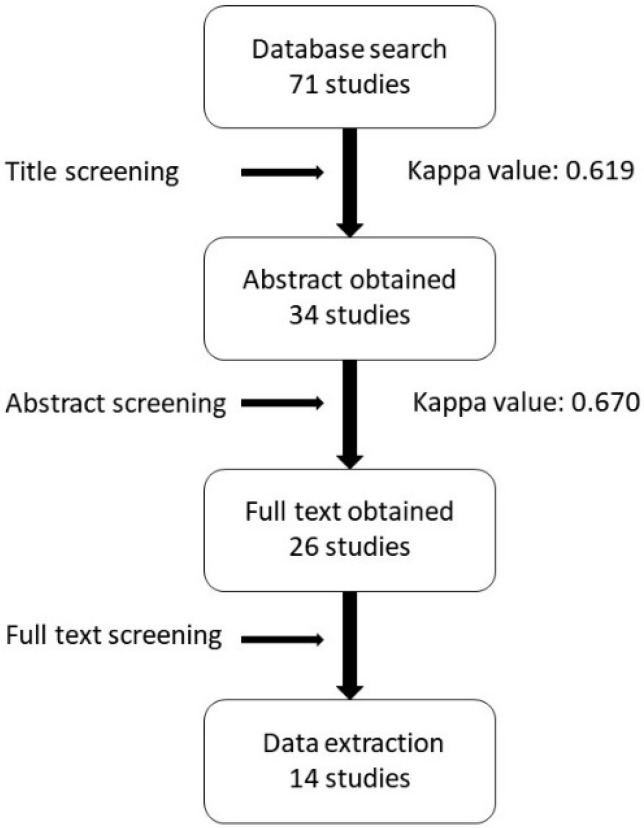
Flowchart of studies selected in the review.

Figure 1 illustrates how 14 studies were selected from the 71 studies initially identified in the electronic search of the period from 2008 to 2018. There were 37 studies excluded from the title screening process for reasons of being reviews, case reports or letters to the editor; reporting on in vitro studies; or being related to early failures only. Screening the Abstracts of the remaining 34 studies resulted in a further 3 articles being excluded for not reporting on implant failures and 5 for not reporting the specific reason for the implant failure. Of the 26 full-text articles assessed by 2 independent reviewers, only 14 studies were finally included in this review [7,12,13,14,15,16,17,18,19,20,21,22,23,24]. Cohen’s kappa values for interexaminer reliability were 0.619 and 0.670 for the title and abstract screening, respectively.

### 3.2. Data Extraction

Table 1 and Table 2 list the risk factors associated with late failure in the 14 studies that fulfilled the inclusion criteria.

#### 3.2.1. Age and Sex

In all of the related studies, neither age nor sex was significantly associated with late failure [7,13,15,16]. However, Manor et al. found that late failure was more common in men than women [7].

#### 3.2.2. Systemic Factors

##### Radiation Therapy

Two studies investigated the effect of radiation therapy on late failure [15,21]: Alsaadi et al. found that radiotherapy significant increased the rate of late implant failure [21], while Doll et al. found that radiochemotherapy patients had a 1.9-fold higher risk of late implant failure compared to ablative surgery patients [15].

##### Diabetes

Controlled diabetes did not significantly influence the late implant failure rate [16,19,20,21].

##### Other Medical Problems

Three articles investigated other medical problems [7,16,21]. Dvorak et al. reported that thyroid disease did not significantly increase the likelihood of late failure [16]. Manor et al. found that a patient with any medical problems had a higher probability of late failure, although this effect was not statistically significant for any specific disease [7]. Alsaadi et al. found that systemic factors did not significantly increase late failures [21].

#### 3.2.3. Oral History

The influence of oral history on late failure was documented in three articles [12,18,20]. Regarding periodontitis, Derks et al. found no associations between a patient with an initial diagnosis of periodontitis and late failure [12]. However, Vercruyssen et al. found that a history of periodontitis was a possible influencing factor for late failure [20]. Moreover, Levin et al. found that while severe periodontitis was not a significant risk factor for late failure up to 50 months postsurgery, it was a significant hazard after 50 months, with a hazard ratio (HR) of 8.06 (*p* < 0.01) [18].

#### 3.2.4. Smoking

Several studies did not find any statistically significant association between late failure and smoking, suggesting that smoking alone does not increase the incidence of late implant failure [12,13,16,17,18,19,20,21]. Noda et al. reported that smokers had a threefold higher risk of late failure than nonsmokers, but this result was not statistically significant [13]. Moreover, Levin et al. reported that smoking did not significantly influence late failure up to 50 months postsurgery, but after 50 months the hazard was almost significant (HR = 2.76, *p* = 0.061) [18].

#### 3.2.5. Implant Location

The impact of localization on late failure was reported in numerous articles, but the results remain controversial. Alsaadi et al. found that implants placed in the maxilla had significantly higher rates of late failure than those placed in the mandible (HR = 2.59, *p* < 0.001) [21]. Moreover, Noda et al. reported that placing an implant in the maxilla was a significant risk factor for late failure (HR = 4.19, *p* = 0.02) [13]. In contrast, different studies by Jemt and colleagues found that placing implants in the mandible significantly increased the rate of late failures, with HR = 2.03 (*p* < 0.05) [23] and HR = 2.63 (*p* < 0.05) [22]. However, numerous studies have found that placing an implant in the maxilla or mandible does not significantly alter the likelihood of late failure [15,16,17].

Two studies indicated that placing an implant in the anterior or posterior region was not a significant risk factor for late failure [16,17]. However, other studies found more late failures in the posterior region than in the anterior region [7,13,21]. Alsaadi et al. found that placing an implant in a posterior location was a significant hazard for late failure compared to an anterior location (HR = 2.14, *p* < 0.001) [21]. Furthermore, Noda et al. reported that placing an implant in a posterior location was a significant risk factor for late failure (HR = 4.18, *p* < 0.01) [13].

#### 3.2.6. Bone Condition

Two studies indicated that the bone status (osteoporosis or osteopenia) was not significantly associated with late failure [16,21]. There were also two studies finding bone resorption to be a significant risk factor for late failure [21,23]. Specifically, Alsaadi et al. reported that bone grade 4 was associated with significantly more late failures than bone grade 2, whereas a lack of bone volume did not significantly affect the late-failure rate [21].

#### 3.2.7. Type of Implant

While no significant association was found between the implant surface treatment and late failure [16,17,21], a machined implant surface [21] and a moderate rough implant surface [16] showed trends toward more late failures. In contrast, Kermalli et al. found that the press-fit implant design with a sintered porous surface (SPS) had a significantly higher late failure than the threaded implant design with a sandblasted–acid-etched (SLA) surface [14].

#### 3.2.8. Implant Length and Diameter

Three studies found no significant association between implant length and late failure [13,20,21]. A study of immediately loaded implants indicated that short implants were significantly associated with late failure (*p* = 0.029) [17].

Most of the studies found that implant diameter was not a significant risk factor for late failure [13,17,23]. Only the study by Alsaadi et al. found a significantly high rate of late failures for wide-platform implants (5 mm) compared with regular (4 mm) and small-diameter (≤3.75 mm) implants [21].

#### 3.2.9. Surgery-Related Factors

All of the four studies related to surgery type found that bone augmentation was not a significant risk factor for late failure [13,16,17,19]. Jemt found that placing more than one implant in a surgical procedure was significantly associated with more implant failures [23]. Alsaadi et al. reported that a high Periotest value, which indicates low implant stability, was a significant risk factor for late failure [21].

Two studies related to the time of implantation produced different results [17,22]: Strietzel et al. found no significant association between the time of implantation and late failure [17], whereas Jemt et al. found that the two-stage surgery was a significant risk factor for late failure [22].

#### 3.2.10. Other Factors

Manor et al. reported that the rate of late failures did not vary significantly between different implant brands; they also found that bruxism did not significantly affect late failures [7]. In contrast, Chrcanovic et al. reported that bruxism was a significant risk factor for late failure [24], while Derks et al. found a significant association between the implant brand and late failure [12]. Jemt et al. found that a diagnosis of inflammation at the implant site during the first year significantly increased the risk of late failure (HR = 17.95, *p* < 0.05) [22]. Noda et al. revealed several significant risk factors for late failure, such as having more than 20 teeth remaining, opposition by a removable partial denture, complete edentulism and a removable denture with the conus-type connection [13].

## 4. Discussion

This review has summarized the significant risk factors for late dental implant failure. It is clear that various local and systemic factors could damage the integrity of the osseointegration established around an implant after occlusal loading. The exposure of an implant and its suprastructure to the oral microbial environment and the application of occlusal forces make detecting the relevant factors more difficult. We propose categorizing the risk factors for late failures that we identified into three groups related to (1) the patient history, (2) clinical parameters and (3) decisions made by the doctor (Table 3). This classification considers the implant treatment workflow processes, from collecting patient information and history-taking to the clinical examination and clinical execution. Recognizing the potential risk factors could help clinicians to alter the treatment plan in individual patients so as to achieve an optimum outcome.

### 4.1. Factors Related to Patient History

#### 4.1.1. Patient Demographics and Medical History

Many patients seeking implant rehabilitation are of an advanced age, which increases the prevalence of systemic medical problems such as diabetes and osteoporosis. These disorders could exert harmful effects on bone metabolism and thereby endanger the integrity of osseointegration. Nonetheless, the results obtained in this review indicate that only radiation therapy could significantly increase the risk of late failure, but not sex, age or medical problems. Radiation therapy (including radiotherapy and radiochemotherapy) could compromise the oral environment so as to significantly increase the risk of late failure [15,21]. A previous review of medically compromised patients performed in 2014 also supported this finding [25].

Regarding the oral history, a history of periodontitis is an important risk factor for late failure. Periodontitis is one of the main reasons for tooth loss that leads to a requirement for implant rehabilitation. Moreover, a previous review indicated that a history of periodontitis could be considered a predictor of peri-implantitis that could lead to late failure [26]. Jemt et al. reported that a history of periodontitis was significantly associated with inflammation at the implant side that could cause peri-implantitis [22]. This effect could be due to the transmission of periodontal pathogens from the teeth to the implant [27]. Only one study found no association between an initial diagnosis of periodontitis and late implant failure [12], but that study only investigated implant loss rather than the peri-implantitis that is one of the indicators of late failure.

It is particularly interesting that two studies found that all subjects with late loss also experienced at least one early loss [22,28]. Although no statistical analysis was performed, clinicians should be mindful of a strong correlation between early and late implant loss when treating patients with a history of early loss.

#### 4.1.2. Habits

Smoking causes several local and systemic diseases and jeopardizes both bone and wound healing processes [29]. Despite all the relevant studies in this review supporting an association between smoking and an increased risk of late failure, the results were not statistically significant. Moreover, the literature supports that smoking significantly affects early failure [30] and exerts a dose-related effect on late failure of dental implant [18]. Thus, clinicians should apply caution toward and adequately inform smoking patients before giving them implant treatment.

Bruxism seems to be as the most important risk factor endangering the implant survival rate [31]. Bruxism is associated with large and unpredictable occlusal forces that could cause various types of complication during implant treatment, including both biologic and mechanical complications such as bone loss around the implant, prosthesis wear or fracture, screw loosening and fixture fracture. Since the prevalence of people with bruxism is common [32], implant treatment on this population is inevitable. Despite numerous studies finding that bruxism had a negative effect on implant outcomes [7,24,33,34,35], we were unable to draw any definitive conclusion about whether or not bruxism is a significant risk factor for late failure. This finding is consistent with a previous review [36] and it could be due to the lack of published studies, smallness of the analyzed samples or lack of bruxism-specific diagnosis methods. Moreover, the close attention paid by clinicians to bruxism patients along with the application of meticulous treatment plans and performing regular follow-ups could reduce the real effect of this parafunction on outcome of dental implant.

### 4.2. Factors Related to Clinical Parameters

#### 4.2.1. Implant Location

Placing an implant in a posterior location was reported as a significant risk factor for late failure [7,13,21], although a few studies have not found a significant association [16,17]. A significant association finding could be due to posterior teeth being subjected to threefold-higher occlusal forces than the anterior teeth [37]. Posterior regions are also known to be at a higher risk of dental plaque accumulation compared to anterior regions [38] and plaque accumulation is associated with gingival inflammation and the initiation of several oral diseases that could lead to failure of dental implant.

Despite implant placement in the maxilla being found to be a significant risk factor for early failure [30], its influence on late failure remains controversial. While most studies have found that whether an implant is placed in the maxilla or mandible does not significantly influence late failure [15,16,17], there are some that have indicated that either the maxilla [21] or the mandible [22,23] could be a risk factor for late failure. Late failure could be associated with implant placement in the maxilla due to the trabecular bone being less dense and the cortical bone being thinner compared to the mandible [39,40]. The weaker bone structure could decrease the initial stability, which is a risk factor for late failure of dental implant [21]. Meanwhile, the reason for the association between late failure and implant placement in the mandible remains unclear.

#### 4.2.2. Bone Condition

The demand for implant placement is higher among elderly women since they are prone to greater osteopenia or osteoporosis. Although these bone metabolic diseases could have a negative impact on implant stability and have shown trends for more late failures, no significant associations between these bone conditions and late failure have been found [16,21]. Neither a lack of bone volume nor the presence of bone dehiscence or fenestration significantly affected the rate of late failures [21]. Moreover, bone augmentation may have a protective effect on implant outcomes, as demonstrated by a significantly lower peri-implantitis rate and a higher survival rate [16].

While the bone condition and bone volume did not significantly influence the rate of late failures of dental implant, a low bone density poses a significant threat to implant outcome. Low bone quality, especially type IV (thin cortical bone with a low density of trabecular bone), was found to significantly increase both the late- and early-failure rates [21,23]. Poor bone quality was reported to be significantly associated with low initial stability [41], which is a significant risk factor for late failure [21]. This finding has also been found in other previous studies [8,42].

#### 4.2.3. Adjacent Dentition

Only one study found that the presence of more than 20 remaining teeth could significantly increase the late failure rate [13]. The reason behind it remains unclear. Therefore, future well-controlled studies are necessary to clarify this finding.

Regarding the opposing dentition to dental implant, a partial or a complete removable denture was reported to be significant risk factors to late failure [13]. The difficulty in occlusion adjustment and the complication of force distribution on the removable denture could contribute to this finding, but an earlier report indicated that opposing dentition was not a risk factor for late failure [43].

Therefore, careful prosthesis planning and meticulous occlusal adjustment are mandatory for the implant long term success.

### 4.3. Factors Related to Decisions Made by the Doctor

#### 4.3.1. Implant Selection

Despite the implant design and surface treatment not significantly influencing late failure of dental implant, there was a tendency for implants with a machined surface to be associated with a higher failure rate [21]. Moreover, a conventional threaded implant (≥10 mm long) with an SLA surface had a more favorable outcome when treating patients with an adequate bone volume, whereas a short press-fit implant (≤7 mm long) with an SPS showed a better outcome in a case of advanced bone resorption (bone height < 5 mm) [14].

It is advisable to place a short implant when the bone height is inadequate. However, this strategy should be performed with caution in immediately loaded implants, for which short implants were associated with a significant decrease in implant success [17]. The reduced bone–implant interface of a short implant may not allow for the sufficient initial stability that is the main requirement of this technique. On the other hand, when using a surgical guide for implant placement, implants longer than 10 mm were likely to have more late failures than shorter implants [44]. This observation is probably due to bone overheating resulting from inadequate coolant irrigation and the accumulation of bone dust while drilling.

Alsaadi et al. found an increased rate of late failure among large-diameter implants, but this could have been due to the surgeon being inexperienced or wide implants usually being employed as “rescue” implants [21]. It should be noted that several studies found that there was no significant correlation between the implant width and late failure [13,17,23].

We found that the implant brand does not appear to significantly influence the late-failure rate, as also found by Manor et al. [7]. In contrast, a subsequent study reported on in 2015 found that a Straumann implant with an SLA surface produced a significantly more favorable outcome than several other rare implant brands (which have not been widely used and even discontinued in the market), such as Biomet 3i, CrescoTi, XiVE, Frialit and Lifecore [12]. Jemt et al. found that the NobelActive conical connection implant recently exhibited a significantly higher late-failure rate than other implant types, but that implant system had been used to treat more-complicated conditions than the other implant systems in that study [22]. It is therefore difficult to interpret these results due to the differences in clinical used and clinician experience among the studies. While it is feasible that the implant brand could impact the late-failure rate of dental implant, we believe that the available evidence indicates there is no significant effect.

The investigations perform in this review suggest that the implant selected does not play an important role in late failure. However, clinicians should pay more attention in certain circumstances such as during implant placement with a surgical guide and immediately loaded implants.

#### 4.3.2. Surgical Procedure

A two-stage surgical protocol has been recommended for implant placement since being introduced by Branemark [1,3]. However, this strategy has been changing to a one-stage, immediate-placement protocol due to its benefit of reducing the treatment time. A study reported on in 2011 found that the time of implantation did not significantly influence the late-failure rate [17]. Nonetheless, it was subsequently found in 2017 that using a two-stage protocol was a significant risk factor for late failure of dental implant, which could be explained by most of the two-stage surgical procedures employing a guided bone regeneration procedure, which indicates that they were complicated cases involving severe bone loss [22].

Several studies have shown that bone augmentation is not significantly associated with late failure. Moreover, implant placement with bone augmentation was found to be likely to have a higher success rate [16]. This protective effect of the bone graft procedure demonstrates that bone grafting is a promising technique for improving the bone quantity without compromising the success rate.

A high Periotest value either during implant insertion or at the abutment connection was found to be a significant risk factor for late failure [21]. This parameter is derived from a method measuring the implant stability, which indicated that clinicians should have a precise surgical plan in order to obtain a high initial stability during implant placement. A higher rate of late failures when placing more than one implant during implant surgery was reported recently [23], which could have been due to the extent of bone loss associated with greater tooth loss, iatrogenic failures due to clinician negligence or fatigue during the extensive surgical intervention.

In addition to the above surgery-related considerations, patient postsurgery follow-up and compliance should also be considered. Most patients report signs of mucosal inflammation or irritation at the implant site before failure actually occurs. An early diagnosis of inflammation at the implant site during the first year was significantly associated with late failure (HR = 17.95) [22]. Thus, patient compliance and implementing a maintenance plan after implant surgery could ensure better outcomes.

#### 4.3.3. Prosthesis Design

An implant-supported overdenture provides several benefits over an implant-fixed prosthesis, such as being cheaper and ease of prosthesis maintenance. However, clinicians should avoid using a conus-type connection, which was reported as a significant risk factor for late failure [13]. Further studies are needed to clarify why such connections are associated with late failure of dental implant.

## 5. Conclusions

This literature review has revealed more risk factors than the previous publication [8]. Based on the results obtained in the present review, the common risk factors related to late failure of dental implant could be classified into three groups including (1) the patient history (radiation therapy, bruxism, periodontitis and early implant loss), (2) clinical parameters (bone grade 4 and implant placed in a posterior location) and (3) decisions made by the doctor (low initial stability, more than one implant placed during surgery or using an implant-supported overdenture with conus-type connection).

The limitation of this literature review is the lack of a data meta-analysis. It is possible that further risk factors could be identified in future well-designed studies.

## Figures and Tables

**Table 1:** Studies that have evaluated the risk factors associated with late dental implant failure (part 1).

Author & Year	No Implants Placed	No Late Failure of Implants Loss	Risk Factors Associated with Late Failure of Dental Implant (Medical History Related)				
			Age & Sex	Systemic History	Oral History	Smoking	Bruxism
Manor et al., 2009 [7]			Age (−)	Medical problem (+)			
Derks et al., 2015 [12]	2367	46 (2%)			Initial diagnosis of periodontitis (−)	(−)	
Noda et al., 2015 [13]	710	10 (1.4%)	Sex (−) Age (−)			(−)	
Doll et al., 2015 [15]	830	20 (2.4%)	Sex (−) Age (−)	RCTx (+)			
Dvorak et al., 2011 [16]	828	69 (8.3%)	Age (−)	Diabetes (−) Thyro (−)		(−)	
Strietzel et al., 2011 [17]	283	5 (1.8%)				(−)	
Levin et al., 2011 [18]	2259	50 (2.2%)			Severe periodontitis (+)	(−)	
Le et al., 2013 [19]	221	1 (0.4%)		Diabetes (−)		(−)	
Vercruyss-en et al., 2010 [20]	1051	37 (3.5%)		Diabetes (−)	Periodontitis (+)	(−)	
Alsaadi et al., 2008 [21]	1514	101 (6.7%)		Diabetes (−) Medical problem (−) RTx (+)		(−)	
Chrcanovic et al., 2017 [24]	854						(+)

(+) statistically significant (*p* < 0.05); (−) not statistically significant (*p* > 0.05); N^o^—Number of; RCTx—radiochemotherapy; RTx—radiotherapy; Thyro—thyroid disease; Bone status is related to osteoporosis/osteopenia.

**Table 2:** Studies that have evaluated the risk factors associated with late dental implant failure (part 2).

Author & Year	N^o^ Implants Placed	N^o^ Late Failure of Implants Loss	Risk Factors Associated with Late Failure of Dental Implant (Implant Treatment Related)					
			Implant Location	Bone Condition	Type of Implant	Implant Size	Surgical Related	Others
Manor et al., 2009 [7]			Posterior (+)					
Derks et al., 2015 [12]	2367	46 (2%)						Implant brand (+)
Noda et al., 2015 [13]	710	10 (1.4%)	Maxilla (+) Posterior (+)			L (−) D (−)	Bone graft (−)	≥20 teeth remaining (+) RD opposing (+) ISO-conus type connection (+)
Kermalli et al., 2014 [14]	799	19 (2.4%)			Press-fit SPS (+)			
Doll et al., 2015 [15]	830	20 (2.4%)	Jaw (−)					
Dvorak et al., 2011 [16]	828	69 (8.3%)	Jaw (−) Posterior (−)	Bone status (−)	Surface treatment (−)		Bone graft (−)	
Strietzel et al., 2011 [17]	283	5 (1.8%)	Jaw (−) Posterior (−)		Surface treatment (−)	Short (+) D (−)	Bone graft (−) Time of implantation (−)	
Le et al., 2013 [19]	221	1 (0.4%)					Bone graft (−)	
Vercruyss-en et al., 2010 [20]	1051	37 (3.5%)				L (−)		
Alsaadi et al., 2008 [21]	1514	101 (6.7%)	Maxilla (+) Posterior (+)	Bone status (−) Bone quality (+)	Surface treatment (−)	L (−) Wide D (+)	High PTVs (+)	
Jemt et al., 2017 [22]	3082	70 (2.3%)	Mandible (+)				2-stages (+)	Inflammation at implant site during 1st year (+)
Jemt et al., 2017 [23]	9582	82 (0.9%)	Mandible (+)	Bone quality (+)		D (−)	N^o^ installed implant at surgery (+)	

(+) statistically significant (*p* < 0.05); (−) not statistically significant (*p* > 0.05); N^o^—Number of; SPS—sintered porous surface; L&D—length and diameter; Wide D—implant with diameter >5 mm; Short—implant shorter than 8 mm; RD—removable denture; ISO—implant-supported overdenture; PTVs—Periotest values.

**Table 3:** Common risk factors associated with late dental implant failure.

Patient History Related				Clinical Parameter-Related		Doctor’s Decision-Related		
Medical History			Habit	Implant Location	Bone Condition	Surgical Procedure		Prosthesis Design
Radiation Therapy	Periodontitis	Early Implant Loss	Bruxism	Posterior	Bone Grade 4	Low Initial Stability	More than 1 Implant Placed at Surgery	Overdenture with Conus-Type Connection
Doll et al. [13]	Levin et al. [16]	Jemt et al. [20]	Manor et al. [5]	Manor et al. [5]	Alsaadi et al. [19]	Alsaadi et al. [19]	Jemt et al. [21]	Noda et al. [11]
Alsaadi et al. [19]	Vercruyssen et al. [18]		Chrcanovic et al. [22]	Noda et al. [11]	Jemt et al. [21]			
	Derks et al. [10]			Alsaadi et al. [19]				
				Dvorak et al. [14]				
				Strietzel et al. [15]				

## References

[1] Branemark P.-I. (1977). Osseointegrated implants in the treatment of the edentulous jaw. Experience from a 10-year period. Scand. J. Plast. Reconstr. Surg. Suppl..

[2] Romeo E., Chiapasco M., Ghisolfi M., Vogel G. (2002). Long-term clinical effectiveness of oral implants in the treatment of partial edentulism: Seven-year life table analysis of a prospective study with ITI^®^ Dental Implants System used for single-tooth restorations. Clin. Oral Implant. Res..

[3] Adell R., Lekholm U., Rockler B., Brånemark P.-I. (1981). A 15-year study of osseointegrated implants in the treatment of the edentulous jaw. Int. J. Oral Surg..

[4] Esposito M., Hirsch J.M., Lekholm U., Thomsen P. (1998). Biological factors contributing to failures of osseointegrated oral implants,(I). Success criteria and epidemiology. Eur. J. Oral Sci..

[5] Tonetti M.S., Schmid J. (1994). Pathogenesis of implant failures. Periodontol. 2000.

[6] Elaskary A. (2008). Fundamentals of Esthetic Implant Dentistry.

[7] Manor Y., Oubaid S., Mardinger O., Chaushu G., Nissan J. (2009). Characteristics of early versus late implant failure: A retrospective study. J. Oral Maxillofac. Surg..

[8] Sakka S., Baroudi K., Nassani M.Z. (2012). Factors associated with early and late failure of dental implants. J. Investig. Clin. Dent..

[9] Clark D., Levin L. (2016). Dental implant management and maintenance: How to improve long-term implant success?. Quintessence Int..

[10] van Steenberghe D., Lekholm U., Bolender C., Folmer T., Henry P., Herrmann I., Higuchi K., Laney W., Lindén U., Åstrand P. (1990). The Applicability of Osseointegrated Oral Implants in the Rehabilitation of Partial Edentulism: A Prospective Multicenter Study on 558 Fixtures. Int. J. Oral Maxillofac. Implant..

[11] Moher D., Liberati A., Tetzlaff J., Altman D.G. (2009). Preferred reporting items for systematic reviews and meta-analyses: The PRISMA statement. J. BMJ.

[12] Derks J., Håkansson J., Wennström J., Tomasi C., Larsson M., Berglundh T. (2015). Effectiveness of implant therapy analyzed in a Swedish population: Early and late implant loss. J. Dent. Res..

[13] Noda T. (2015). A longitudinal retrospective study of the analysis of the risk factors of implant failure by the application of generalized estimating equations. J. Prosthodont. Res..

[14] Kermalli J.Y., Deporter D.A., Atenafu E.G., Lam E.W. (2014). A retrospective report on three implant devices used to restore posterior partial edentulism: Overall performance and changes in crestal bone levels. Int. J. Periodontics Restor. Dent..

[15] Doll C., Nack C., Raguse J.-D., Stricker A., Duttenhoefer F., Nelson K., Nahles S. (2015). Survival analysis of dental implants and implant-retained prostheses in oral cancer patients up to 20 years. Clin. Oral Investig..

[16] Dvorak G., Arnhart C., Heuberer S., Huber C.D., Watzek G., Gruber R. (2011). Peri-implantitis and late implant failures in postmenopausal women: A cross-sectional study. J. Clin. Periodontol..

[17] Strietzel F.P., Karmon B., Lorean A., Fischer P.P. (2011). Implant-prosthetic rehabilitation of the edentulous maxilla and mandible with immediately loaded implants: Preliminary data from a retrospective study, considering time of implantation. Int. J. Oral Maxillofac. Implant..

[18] Levin L., Ofec R., Grossmann Y., Anner R. (2011). Periodontal disease as a risk for dental implant failure over time: A long-term historical cohort study. J. Clin. Periodontol..

[19] Le B.T., Follmar T., Borzabadi-Farahani A. (2013). Assessment of short dental implants restored with single-unit nonsplinted restorations. Implant Dent..

[20] Vercruyssen M., Marcelis K., Coucke W., Naert I., Quirynen M. (2010). Long-term, retrospective evaluation (implant and patient-centred outcome) of the two-implants-supported overdenture in the mandible. Part 1: Survival rate. Clin. Oral Implant. Res..

[21] Alsaadi G., Quirynen M., Komárek A., Van Steenberghe D. (2008). Impact of local and systemic factors on the incidence of late oral implant loss. Clin. Oral Implant. Res..

[22] Jemt T., Karouni M., Abitbol J., Zouiten O., Antoun H. (2017). A retrospective study on 1592 consecutively performed operations in one private referral clinic. Part II: Peri-implantitis and implant failures. Clin. Implant Dent. Relat. Res..

[23] Jemt T. (2017). A retro-prospective effectiveness study on 3448 implant operations at one referral clinic: A multifactorial analysis. Part II: Clinical factors associated to peri-implantitis surgery and late implant failures. Clin. Implant Dent. Relat. Res..

[24] Chrcanovic B.R., Kisch J., Albrektsson T., Wennerberg A. (2017). Bruxism and dental implant treatment complications: A retrospective comparative study of 98 bruxer patients and a matched group. Clin. Oral Implant. Res..

[25] Gómez-de Diego R. (2014). Indications and contraindications of dental implants in medically compromised patients: Update. Med. Oral Patol. Oral Cir. Bucal.

[26] Heitz-Mayfield L.J. (2008). Peri-implant diseases: Diagnosis and risk indicators. J. Clin. Periodontol..

[27] Karoussis I.K., Kotsovilis S., Fourmousis I. (2007). A comprehensive and critical review of dental implant prognosis in periodontally compromised partially edentulous patients. Clin. Oral Implant. Res..

[28] Koldsland O.C., Scheie A.A., Aass A.M. (2009). Prevalence of implant loss and the influence of associated factors. J. Periodontol..

[29] Porter S.E., Hanley E.N. (2001). The musculoskeletal effects of smoking. JAAOS J. Am. Acad. Orthop. Surg..

[30] Manzano G., Montero J., Martín-Vallejo J., Del Fabbro M., Bravo M., Testori T. (2016). Risk factors in early implant failure: A meta-analysis. Implant Dent..

[31] De Angelis F., Papi P., Mencio F., Rosella D., Di Carlo S., Pompa G. (2017). Implant survival and success rates in patients with risk factors: Results from a long-term retrospective study with a 10 to 18 years follow-up. Eur. Rev. Med. Pharmacol. Sci..

[32] Mohl N.D. (1988). A Textbook of Occlusion.

[33] Yadav K., Nagpal A., Agarwal S., Kochhar A. (2016). Intricate Assessment and Evaluation of Effect of Bruxism on Long-term Survival and Failure of Dental Implants: A Comparative Study. J. Contemp. Dent. Pract..

[34] Ji T.-J., Kan J.Y., Rungcharassaeng K., Roe P., Lozada J.L. (2012). Immediate loading of maxillary and mandibular implant-supported fixed complete dentures: A 1-to 10-year retrospective study. J. Oral Implantol..

[35] Glauser R., Ree A., Lundgren A., Gottlow J., Hammerle C.H., Scharer P. (2001). Immediate occlusal loading of Brånemark implants applied in various jawbone regions: A prospective, 1-year clinical study. Clin. Implant Dent. Relat. Res..

[36] Chrcanovic B.R., Albrektsson T., Wennerberg A. (2015). Bruxism and dental implants: A meta-analysis. Implant Dent..

[37] Helkimo E., Carlsson G.E., Helkimo M. (1977). Bite force and state of dentition. Acta Odontol. Scand..

[38] Sreenivasan P.K., Prasad K.V. (2017). Distribution of dental plaque and gingivitis within the dental arches. J. Int. Med. Res..

[39] Park H.-S., Lee Y.-J., Jeong S.-H., Kwon T.-G. (2008). Density of the alveolar and basal bones of the maxilla and the mandible. Am. J. Orthod. Dentofac. Orthop..

[40] Chugh T., Ganeshkar S.V., Revankar A.V., Jain A.K. (2013). Quantitative assessment of interradicular bone density in the maxilla and mandible: Implications in clinical orthodontics. Prog. Orthod..

[41] Turkyilmaz I., McGlumphy E.A. (2008). Influence of bone density on implant stability parameters and implant success: A retrospective clinical study. BMC Oral Health.

[42] Sakka S., Coulthard P. (2009). Bone quality: A reality for the process of osseointegration. Implant Dent..

[43] Chung D.M., Oh T.-J., Lee J., Misch C.E., Wang H.-L. (2007). Factors affecting late implant bone loss: A retrospective analysis. Int. J. Oral Maxillofac. Implant..

[44] Yong L.T., Moy P.K. (2008). Complications of Computer-Aided-Design/Computer-Aided-Machining-Guided (NobelGuide™) Surgical Implant Placement: An Evaluation of Early Clinical Results. Clin. Implant Dent. Relat. Res..

